# How to Assess Perceived Risks and Safety Behaviors Related to Pandemics: Developing the Pandemic Risk and Reaction Scale during the Covid-19 Outbreak

**DOI:** 10.18502/ijps.v15i4.4293

**Published:** 2020-10

**Authors:** Mohammad Reza Mohammadi, Hadi Zarafshan, Sahar Khayam Bashi, Ali Khaleghi

**Affiliations:** 1 Psychiatry and Psychology Research Center, Tehran University of Medical Sciences, Tehran, Iran.; 2 Department of Autism and Neurodevelopmental Disorders, Psychiatry and Psychology Research Center, Tehran University of Medical Sciences, Tehran, Iran.

**Keywords:** *Pandemic*, *Risk Perception*, *Safety Behaviors*, *Scale*

## Abstract

**Objective:** The aim of the present study was to develop a self-report questionnaire to assess the level of perceived risks and safety behaviors during pandemics.

**Method** **:** We went through recommended phases and their corresponding steps to create a valid and reliable scale: (a) item development (including 1. domain identification and item generation, 2. content validity), (b) scale development (including 1. pretesting questions, 2. sampling and survey administration, 3. item reduction, and 4. extraction of factors), and (c) scale evaluation (including 1. tests of dimensionality, 2. tests of reliability, and 3. tests of validity).

**Results: **We found four factors with eigenvalues greater than 1 that were accounted for 0.63 of the total variance. The 4-factor solution showed all items had factor loading greater than 0.4 and each belonged to one factor. The fit indices indicated the 4-factor solution model was fitted to our data.

**Conclusion: **In sum, the Pandemic Risk and Reaction Scale (PRRS) is a valid and reliable self-reported scale to assess the level of perceived risk and safety behaviors during pandemics.

Adopting appropriate safety behaviors is the best known way to control pandemics like new corona virus (COVID-19) (1, 2). Safety behaviors include 2 broad categories; ie, avoidant behaviors (eg, staying at home, avoiding public transportation) and preventive behaviors (eg, washing hands, using sanitizers) (3). Based on the current evidences from past pandemics, the level of both avoidant and preventive behaviors is related to some personal factors. Being female, older age, having small children at home, and higher educational degrees increase the level of safety behaviors (4-6). 

Risk perception is an important concept which assumed to be the core predictor of safety behaviors in different health theories. “Risk perception” or “perceived risk” refers to one’s judgment in regards to the consequences of a harmful event like pandemics (5, 7). 

Both inadequate or excessive level of perceived risk are problematic; low level of contributed risk significantly decreases the likelihood of following necessary safety protocols and high level of contributed risk increases the proportion of mental health problems including anxiety, stress, and depression (4, 8, 9). For example, in a telephone-based survey during the second wave of N1H1 influenza in the United States, it was revealed that public risk perception was significantly associated with preparatory behaviors (10). On the other side, some experimental studies have shown that health related safety behaviors are positive predictors of high level of health anxiety (11). Also, it is found that excessive level of perceived risk elaborates unnecessary safety behaviors that have significant negative consequences on personal life (12).

Risk perception and safety behaviors are correlated variables and their optimum level is crucial in management of any pandemic. Some researchers have developed self-report scales to assess responses to previous (eg, N1H1) and new (ie, corona virus) pandemics and published their psychometric findings (13-15). For example, the Fear of COVID-19 Scale (FCV-19S), a unidimensional 7-item scale, is developed to assess fear responses to the corona virus pandemic and is validated in different cultures (16). Although some of the published scales have had good psychometric properties, most of them have focused on psychological reactions, including fear, stress and anxiety related to the pandemic, and actual behavioral responses are left behind.

Given that the risk perception and safety behaviors are important variables in pandemic studies, we have developed a brief self-reported questionnaire to assess these concepts simultaneously among general population. In this paper we have presented the procedure of scale development including item generation, explanatory and confirmatory factor analysis. We named this questionnaire the Pandemic Risk and Reaction Scale (PRRS).

## Materials and Methods

***Procedure***

We went through recommended phases to create a valid and reliable scale: (a) item development (including 1. domain identification and item generation, 2. content validity), (b) scale development (including 1. pretesting of questions, 2. sampling and survey administration, 3. item reduction, and 4. extraction of factors), and (c) scale evaluation (including 1. tests of dimensionality, 2. tests of reliability, and 3. tests of validity).

At first, we comprehensively reviewed current theories of risk communication, risk perception, and safety behaviors, and existing evidences from previous pandemics. We carefully discussed obtained data and finally concluded 2 main categories for risk perception (ie, societal risk and personal risk) and 2 main categories for safety behaviors (ie, avoidant behaviors and preventive behaviors). The “societal risk” refers to one’s perception of how an event can be hazardous for entire society and “personal risk” refers to perceived hazard about oneself. “Avoidant behavior” refers to a group of behaviors like staying at home and avoiding public transportation. “Preventive behavior” refers to a group of behaviors like washing hands and using sanitizers.

Then, we generated items corresponding to above mentioned categories. Some items were modified versions of questions asked in previous surveys during the pandemics and some of them were new. Initially, we developed a set of 19 items and then reduced them to 15 items during our group discussions. 

To assess content validity, we sent the 15-item questionnaire to a group of 5 experts in the fields of psychiatry, psychology, and sociology and wanted them to rate the relevancy of each item on a Likert scale from 1 to 5 and then calculated Cohen’s coefficient kappa (k). The k values showed the agreement between raters were in perfect range for all items (They were more than 0.90.). Then, we sent our questionnaire to a sample of respondents and asked them to rate each item on a Likert scale from 1 to 5, which reflected their understandability. Next, we checked pilot respondents’ thoughts about each item using cognitive interview. All items were confirmed and we finalized a 15-item questionnaire.

***Data Collection***

To increase the speed of data gathering and avoid the risk of disease transition, we decided to use online administration method of data collection. We used a local commercial web-based platform and developed an online version of the questionnaire. Using convenient and snow-ball sampling method, we distributed the link of online questionnaire by different media and asking the receiver to share the link.

***Sampling***

As this scale was developed to assess the perceived risks and safety behaviors among general population, our sampling frame was all Iranian citizens who potentially were able to receive and respond to the questionnaire.

Although there is controversy in sample size estimation in scale development studies, a sample size of around 1000 respondents is considered as excellent. Also, it is highly recommended to use 2 independent samples; one group for primary scale development and another for confirmatory factor analysis. Hence, we considered the sample size equal to 2.000; ie, 2 sets of 1000 respondents. We ran primary analysis on the first set of 1000 respondents and then confirmatory analysis on the second set of 1000 respondents.

***Scales***

***The Pandemic Risk and Reaction Scale (PRRS)***

We developed a self-reported scale. The PRRS has 15 items that measure perceived risk and safety behaviors during the pandemic. It also has 4 subscales including societal risk (4 items), personal risk (3 items), avoidant behavior (5 items), and preventive behavior (3 items). Each item will be rated on a 5-point Likert scale, ranging from 1 to 5. This scale presents 4 distinct mean scales for each subscale. 

***The 28-item General Health Questionnaire (GHQ-28)***

The GHQ-28 has 4 subscales, each has 7 items that measure symptoms of somatization, anxiety, social dysfunction, and depression. In this study we used the traditional scoring method (giving 0-0-1-1) with a cutoff equal to 6 for the total score to assess discriminant validity. Based on standardization study among Iranian population, the cutoff score of 6 has 84.2% sensitivity and 94.4% specificity. We also used 0-1-2-3 coding method of each item to compute total score and assess convergent validity (17, 18).

***Short Health Anxiety Inventory (SHAI)***

The SHAI contains 18 items that assess health anxiety independent from current health status. Items assess worrying about health, awareness of bodily sensations or changes, and feared consequences of having an illness. The SHAI has demonstrated good reliability, criterion validity, and sensitivity to treatment. The Cronbach’s of this scale was reported as 0.78 among Iranian sample (19, 20). We used the mean of the total score in our study to assess convergent validity.

***Kessler Psychological Distress Scale (K10)***

The K10 is a 10-item questionnaire to assess psychological distress based on questions about anxiety and depressive symptoms that a person has experienced in the past 4-week period. Each item will be rated on a 5-point Likert scale, ranging from 1 to 5. Total scores will range from 10 to 50 (21). We converted the total score to 2 categories. “Low level of psychological distress” (score 10 to 21), and “high level of psychological distress” (scores 22 to 50). Cronbach’s alpha and Spearman-Brown coefficients of the K10 reached 0.92 and split-half 0.85, showing a good internal consistency among Iranian population. In this study we used the mean of the total score to assess convergent validity and 2-level symptoms for discriminant validity. 

***Impact of Event Scale - Revised (IES-R)***

The IES-R is a 22-item self-report measure to assesses subjective distress caused by traumatic events. Items are rated on a 5-point scale ranging from 0 to 4 (22, 23). In IES-R we can use mean of responses instead of sum of responses which is in the same metric as the item responses (24). The mean score equal to 1.5 is considered as best cut off point to diagnose who have high level of event related stress symptoms (acute stress symptoms) and are at high risk to develop post-traumatic stress disorders in future (25). The Persian version of IES-R has shown good internal consistency (Cronbach Alpha = 0.67-0.87) and test-retest reliability (r = 0.8-0.98, P < 0.001) and also good convergent validity. In present study we used the mean of the total score to assess convergent validity and 2-level symptoms for discriminant validity.

***Data Analysis***

We used descriptive statistics to understand participants’ characteristics. To control the effects of sampling error, we ran principal component analysis (PCA) on the first set of 1000 respondents and then performed confirmatory factor analyses (CFAs) on the second set of 1000 respondents. We used the standardized-root-mean- square residual (SRMR), the root-mean-square error of approximation (RMSEA), and the comparative fit index (CFI) as goodness-of-fit indices. We used Cronbach’s coefficient alpha to assess internal consistency. We also ran a series of correlational analyses, the analysis of variance (ANOVA test), and one sample t test to assess convergent validity and discriminant validity. All statistical analyses were performed by STATA version 14.

## Results

***Sample characteristics***

Table 1 shows sample characteristics for explanatory and confirmatory phases separately. In both samples, females and married persons were dominant. Most respondents were between 31 to 60 years old and had bachelor or master degrees.

***Principal component analysis***

At first, we checked the factorability of the data. The Kaiser-Meyer-Olkin Measure of Sampling Adequacy was acceptable (KMO = 0.81) and the p-value of Bartlett test of sphericity was < 0.001. These results confirmed that our data were appropriate for factor analysis.

**Figure 1:**
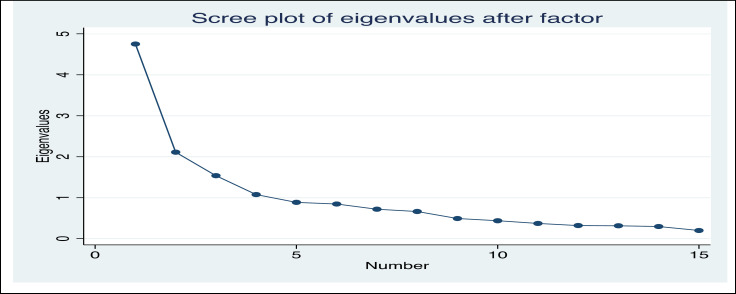
Scree Plot of Factors with Eigenvalues Greater than 1

We used principal-component factors (pcf) and “promax” rotation for item reduction and factor extraction. As seen in “screeplot” (figure 1), we found 4 factors with eigenvalues greater than 1 that were accounted for 0.63 of the total variance. The 4-factor solution showed that all items have had factor loading greater than 0.4 and each belonged to one factor. The identified factors and related items were exactly the same as what we have generated based on the literature, including societal risk, personal risk, avoidant behaviors, and preventive behaviors. Factor correlation matrix showed all factors were positively correlated; however, they did not exceed critical value of 0.7.

Based on the Cronbach Alpha values, the level of internal consistency for total items (□=0.83) and each subfactors (□=0.86, □=0.74, □=0.70 and □=0.78 for factors 1, 2, 3 and 4, respectively) were in acceptable range. Also, both item-rest correlation and item-test correlation showed that all items were correlated (see table 2). 

***Confirmatory factor analysis***

The 4-factor model obtained in the explanatory phase from the first set of 1000 respondents was checked by confirmatory factor analysis on the second set of 1000 respondents. The results of Kaiser-Meyer-Olkin Measure of Sampling Adequacy (KMP = 0.82) and the p-value of Bartlett test of sphericity (p < 0.001) showed that the second set of data was also appropriate for analysis. 

As seen in Table 3, all items had acceptable factor loadings. Also, total items and subscales had good internal consistencies (see alpha coef. and item-rest correlation and item-test correlation). 

The fit indices indicated that the 4-factor solution model was fitted to our data. All of the goodness-of-fit indices are presented at Table 4. 

***Convergent and discriminant validity***

**Figure 2:**
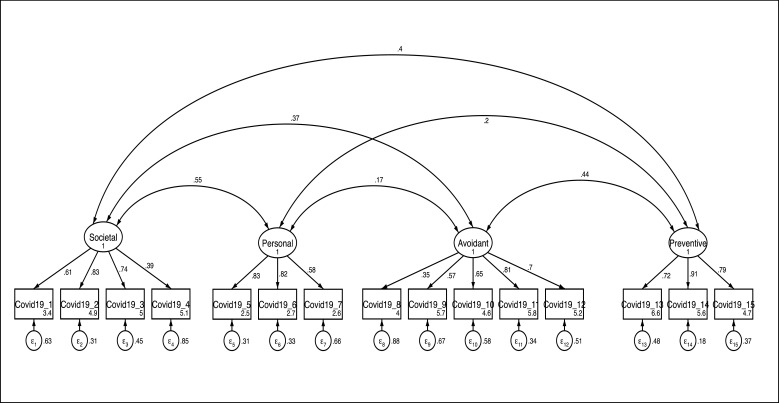
Four Factor Model of Pandemic Risk and Reaction Scale (PRRS)

We checked convergent and discriminant validity after confirmatory factor analysis based on data obtained from second 1000 respondents. Figure 2 shows that all factors are positively correlated, which can be considered as an index of convergent validity. Furthermore, we checked the correlation between total scores of SHAI, IES, and GHQ with each subfactors of our scales. As seen in Table 5, all variables had a significant positive correlation.

To assess discriminant validity of our scale, we ran other set of statistical analysis. As we assumed, a single sample t test revealed that the mean score of societal risk is significantly higher than personal risk (t (999) = 45.35, p < 0.001). Also, the analysis of variance (ANOVA test) showed that the mean of subfactors in our scale can be significantly different based on some variables (see table 6). For example, the mean of societal risk is significantly higher among the females, the persons who feel symptoms of covid-19 in themselves or their relatives, the persons who have positive cases in their relatives, and the persons who fall in problematic level of psychological symptoms based on the total scores in K-10, IES and GHQ.

**Table 1:** Descriptive Data of Total Study Participants

	**Explanatory phase sample**		**Confirmatory phase sample**	
**Variable**	**N**	**Percent**	**N**	**Percent**
Total sample	1,000	100	1,000	100
Sex				
Male	365	36.5	333	33.3
Female	635	63.5	667	66.7
Age				
Under 20 years	20	2	28	2.8
21-30	197	19.7	213	21.3
31-40	427	42.7	377	37.7
41-50	228	22.8	223	22.3
51-60	97	9.7	130	13
61+	31	3.1	29	2.9
Education level				
Primary school	0	0	6	0.6
Guidance school	12	1.2	22	2.2
High school	8	0.8	25	2.5
Diploma	109	10.9	118	11.8
Post-Diploma	44	4.4	67	6.7
Bachelor degree	323	32.3	332	33.2
Master Degree	303	30.3	275	27.5
PhD +	201	20.1	155	15.5
Marital Status				
Single	326	32.6	326	32.6
Married	674	67.4	674	67.4
Living Alone				
No	921	92.1	912	91.2
Yes	79	7.9	88	8.8
Feeling covid-19 symptoms in past 2 weeks				
No	814	81.4	798	79.8
Yes	186	18.6	202	20.2
Feeling covid-19 symptoms among relatives and friends in past 2 weeks				
No	806	80.6	787	78.7
Yes	194	19.4	213	21.3
Positive cases in relatives and friends				
No	897	89.7	871	87.1
Yes	103	10.3	129	12.9
Having high risk medical condition				
No one	757	75.78	771	77.1
Heart disease	45	4.5	38	3.8
Kidney disease	17	1.7	11	1.1
Diabetes	36	3.6	37	3.7
Gastrointestinal disease	83	8.31	85	8.5
Respiratory and lung disease	61	6.11	58	5.8

**Table 2:** Summary of Principal Component Analysis among the First Set of 1000 Respondents

**Subscales**	**Variable**	**Mean**	**Std. Dev.**	**item-test correlation**	**item-rest correlation**	**alpha**		**Factor1**	**Factor2**	**Factor3**	**Factor4**	**Alpha**
Societal risk	Covid19_1	3.64	1.08	0.65	0.55	0.82				0.40		
	Covid19_2	4.22	0.88	0.64	0.57	0.82				0.87		0.71
	Covid19_3	4.28	0.84	0.60	0.53	0.82				0.88		
	Covid19_4	4.37	0.80	0.41	0.31	0.83				0.53		
Personal risk	Covid19_5	2.71	1.04	0.55	0.44	0.83					0.89	
	Covid19_6	3.04	1.08	0.54	0.43	0.83					0.85	0.78
	Covid19_7	2.95	1.04	0.54	0.44	0.83					0.76	
	Covid19_8	4.41	1.15	0.46	0.33	0.84			0.44			
Avoidant behavior	Covid19_9	4.73	0.79	0.49	0.41	0.83			0.84			0.74
	Covid19_10	4.60	0.92	0.53	0.44	0.83			0.87			
	Covid19_11	4.68	0.71	0.59	0.53	0.82			0.76			
	Covid19_12	4.58	0.82	0.59	0.51	0.82			0.55			
Preventive behavior	Covid19_13	4.64	0.68	0.56	0.49	0.82		0.87				
	Covid19_14	4.50	0.80	0.59	0.51	0.82		0.97				0.87
	Covid19_15	4.33	0.96	0.59	0.50	0.82		0.90				
						0.83						
							Factor1	1				
							Factor2	0.41	1			
					Factor correlation matrix		Factor3	0.39	0.35	1		
							Factor4	0.22	0.22	0.43	1	

**Table 3:** Summary of Confirmatory Factor Analysis among the Second Set of 1000 Respondents

	**Variable**	**Mean**	**Std. Dev.**	**item-test ** **correlation**	**item-rest ** **correlation**	**alpha**	**Factor ** **loading**	**Std.Err.**	**z**	**P>z**	**95% Conf.** **Interval**		**Alpha**
											Lower	Upper	
Societal risk	Covid19_1	3.64	1.06	0.63	0.53	0.81	0.61	0.02	24.90	0.00	0.56	0.65	
	Covid19_2	4.27	0.88	0.62	0.54	0.81	0.83	0.02	47.98	0.00	0.80	0.86	0.72
	Covid19_3	4.29	0.86	0.57	0.49	0.81	0.74	0.02	38.80	0.00	0.70	0.78	
	Covid19_4	4.29	0.85	0.41	0.31	0.82	0.39	0.03	12.71	0.00	0.33	0.45	
Personal risk	Covid19_5	2.70	1.09	0.54	0.43	0.81	0.83	0.02	47.40	0.00	0.80	0.87	
	Covid19_6	3.02	1.11	0.56	0.45	0.81	0.82	0.02	45.95	0.00	0.78	0.85	0.78
	Covid19_7	2.97	1.13	0.53	0.41	0.82	0.58	0.03	23.66	0.00	0.53	0.63	
Avoidant behavior	Covid19_8	4.48	1.13	0.44	0.31	0.82	0.35	0.03	11.20	0.00	0.29	0.41	
	Covid19_9	4.70	0.82	0.49	0.40	0.82	0.57	0.03	22.40	0.00	0.52	0.62	0.73
	Covid19_10	4.55	0.99	0.51	0.41	0.82	0.65	0.02	28.68	0.00	0.61	0.70	
	Covid19_11	4.62	0.80	0.56	0.48	0.81	0.81	0.02	45.96	0.00	0.78	0.85	
	Covid19_12	4.56	0.88	0.53	0.44	0.81	0.70	0.02	33.54	0.00	0.66	0.74	
Preventive behavior	Covid19_13	4.63	0.70	0.54	0.47	0.81	0.72	0.02	39.52	0.00	0.68	0.76	
	Covid19_14	4.51	0.81	0.59	0.51	0.81	0.91	0.01	70.27	0.00	0.88	0.93	0.84
	Covid19_15	4.37	0.92	0.60	0.51	0.81	0.79	0.02	51.12	0.00	0.76	0.82	

**Table 4:** Fit Indices of 4-Factor Solution Model of the Pandemic Risk and Reaction Scale

**Fit statistic**	**Value**	**Description**
Likelihood ratio		
chi2_ms(84)	355.513	model vs. saturated
p > chi2	0.000	
chi2_bs(105)	5,263.113	baseline vs. saturated
p > chi2	0.000	
Population error		
RMSEA	0.057	Root mean squared error of approximation
90% CI, lower bound	0.051	
upper bound	0.063	
pclose	0.031	Probability RMSEA <= 0.05
Information criteria		
AIC	35,392.970	Akaike's information criterion
BIC	35,643.265	Bayesian information criterion
Baseline comparison		
CFI	0.947	Comparative fit index
TLI	0.934	Tucker-Lewis index
Size of residuals		
SRMR	0.048	Standardized root mean squared residual
CD	0.999	Coefficient of determination

**Table 5:** Correlation between Subfactors of the Pandemic Risk and Reaction Scale and Total Scores of Psychological Scales

**Variable**	**N**	**Mean**	**Std. Dev.**	**Societal risk**	**Personal risk**	**Avoidant behavior**	**Preventive behavior**
SHAI total	1,000	0.840	0.439	0.351	0.395	0.150	0.202
				0.000	0.000	0.000	0.000
IES total	1,000	1.535	0.717	0.398	0.368	0.152	0.235
				0.000	0.000	0.000	0.000
K-10_total	1,000	20.250	8.272	0.336	0.338	0.062	0.129
				0.000	0.000	0.050	0.000
GHQ total	1,000	23.962	12.357	0.376	0.380	0.122	0.162
				0.000	0.000	0.000	0.000

Short Health Anxiety Inventory (SHAI), Impact of Event Scale - Revised (IES-R), Kessler Psychological Distress Scale (K10), The 28-item General Health Questionnaire (GHQ-28)

**Table 6:** Relationship between the Pandemic Risk and Reaction Scale Subfactors and Other Variables as Index of Discriminant Validity

		**Societal risk**		**Personal risk**		**Avoidant ** **behavior**		**Preventive ** **behavior**	
**variable**	**comparison**	**ANOVA**	**Bonferroni**	**ANOVA**	**Bonferroni**	**ANOVA**	**Bonferroni**	**ANOVA**	**Bonferroni**
			mean difference		mean difference		mean difference		mean difference
Sex	Female – Male	f (1,998)=9.76, p<0.01	MD=0.14, p<0.01	f (1,998)=8.14, p<0.01	MD=-0.17, p<0.01	f (1,998)=13.07, p<0.001	MD=0.15, p<0.001	f (1,998)=7.85, p<0.01	MD=0.13, p<0.01
Feeling covid- 19 symptoms in past 2 weeks	Yes-No	f (1,998)=20.31, p<0.001	MD=0.23, p<0.001	f (1,998)=45.25, p<0.001	MD=0.47, p<0.001	f (1,998)=0.2, p=0.88	MD=0.007, p=0.88	f (1,998)=3.66,p<0. 05	MD=0.10, p<0.05
Feeling covid- 19 symptoms among relatives and friends in past 2 weeks	Yes-No	f (1,998)=17.63, p<0.001	MD=0.21, p<0.001	f (1,998)=49.73, p<0.001	MD=0.50, p<0.001	f (1,998)=2.27, p=0.13	MD=0.07, p=0.13	f (1,998)=2.79, p=0.09	MD=0.09, p=0.09
Positive cases in relatives and friends	Yes-No	f (1,998)=10.84, p<0.01	MD=-0.20, p<0.01	f (1,998)=37.49, p<0.001	MD=0.52, p<0.001	f (1,998)=0.31, p=0.58	MD=0.03, p=0.58	f (1,998)=0.03, p=0.85	MD=-0.01, p=0.85
IES total	High-Low	f (1,998)=121.14, p<0.001	MD=0.44, p<0.001	f (1,998)=96.41, p<0.001	MD=0.54, p<0.001	f (1,998)=14.73, p<0.001	MD=0.15, p<0.001	f (1,998)=47.45, p<0.001	MD=0.30, p<0.001
K-10 total	High-Low	f (1,998)=92.21, p<0.001	MD=0.40, p<0.001	f (1,998)=103.81, p<0.001	MD=0.60, p<0.001	f (1,998)=0.98, p=0.32	MD=0.04, p=0.32	f (1,998)=8.72, p<0.01	MD=0.13, p<0.01
GHQ total		f (1,998)=138.92, p<0.001	MD=0.48, p<0.001	f (1,998)=124.79, p<0.001	MD=0.63, p<0.001	f (1,998)=17.39, p<0.001	MD=0.17, p<0.001	f (1,998)=15.85, p<0.001	MD=0.18, p<0.001

Impact of Event Scale - Revised (IES-R), Kessler Psychological Distress Scale (K10), The 28-item General Health Questionnaire (GHQ-28)

## Discussion

The aim of the present study was to develop a self-report questionnaire to assess the level of perceived risks and safety behaviors during pandemics. Based on the literature, perceived risk during the disasters consists of “societal risk” and “personal risk” and safety behavior includes “avoidant behaviors” and “preventive behaviors”. We developed a 15-item scale, the Pandemic Risk and Reaction Scale (PRRS), which has 4 items for “societal risk”, 3 for “personal risk”, 5 for “avoidant behavior” and 3 for “preventive behavior”. We found a 4-factor solution using principal component analysis, which confirmed by confirmatory factor analysis. The scale also shows good reliability and validity.

Previous studies have shown a positive relationship between perceived risk and problematic mental health symptoms during disasters. As an index of convergent validity, we found that the mean of PRRS subscales are positively related to the total scores of SHAI, K-10, IES, and GHQ. It is also revealed that the mean of PRRS subscales are significantly different between those who have high level of psychological symptoms and those with low level of psychological symptoms. Previous scales have mainly focused on psychological responses during pandemics.

***PRRS***

As mentioned previously, another new questionnaire which is developed to assess psychological responses to covid-19 is the Fear of COVID-19 Scale (FCV-19S). The FCV-19S was primarily developed and assessed among Iranian population and then used in different countries (16, 26, 27). As we found in our study, there is a positive correlation between FCV-19S sore and the level of mental health problems in general population. Although the FCV-19S have shown good psychometric properties, it only focused on fear responses. The PRRS went further and assessed societal risk (4 items), personal risk (3 items), avoidant behavior (5 items), and preventive behavior (3 items) simultaneously. The PRRS allows assessing more areas, while it keeps briefness and does not have many more items.

The COVID Stress Scales (CSS) is another self-reported questionnaire (28). It has 35 items and assessed 5 different areas of COVID-related stress and anxiety symptoms: (1) Danger and contamination fears, (2) fears about economic consequences, (3) xenophobia, (4) compulsive checking and reassurance seeking, and (5) traumatic stress symptoms. A large population-based study has shown that CSS has good psychometric properties and its scores are positively correlated with problematic psychological symptoms like obsessive-compulsive disorder (OCD), anxiety, depression, xenophobia, and health anxiety. Although a good characteristic of CSS is that it covers important areas like “fears about economic consequences” and “traumatic stress symptoms”, it focused on psychological reactions, not actual behaviors. Another issue about CSS is that it is relatively a long questionnaire and takes much time to fill out.

## Limitation

The main limitation of our study is data gathering and sampling method (ie, online survey with snowball sampling method,) which may have affected our findings. However, we had to use these methods due to the dangerous situation of the pandemic.

## Conclusion

In sum, the Pandemic Risk and Reaction Scale (PRRS) is a valid and reliable self-reported questionnaire to assess the level of psychological responses and actual behavioral reactions simultaneously during pandemics.

Also, this scale can be used to distinguish between those who experience high and low levels of health anxiety and acute stress during pandemics.

**Appendix:** The Pandemic Risk and Reaction Scale (PRRS) Subscales and Items

**Subscales**	**Items**	**A ** **little**				**Very ** **much**
Societal Risk	1. To what extent do you worry about “…”?	1	2	3	4	5
	2. In your opinion, to what extent can “…” become an epidemy in your country?	1	2	3	4	5
	3. In your opinion, how fast “…” will spread in your country?	1	2	3	4	5
	4. To what extent do you seek information about “…”?	1	2	3	4	5
Personal Risk	5. How likely do you think you will be infected by “…”?	1	2	3	4	5
	6. How likely do you think one of your relatives/friends will be infected by “…”?	1	2	3	4	5
	7. In your opinion, how severe will the symptoms be if you get “…”?	1	2	3	4	5
Avoidant Behaviors	8. To what extent your travel plans have affected by the risk of “…”?	1	2	3	4	5
	9. To what extent the risk of “…” has prevented you from eating outside the home?	1	2	3	4	5
	10. To what extent the risk of “…” has prevented you to use of public transportation?	1	2	3	4	5
	11. To what extent the risk of “…” has prevented you to visit/be with public places?	1	2	3	4	5
	12. To what extent the risk of “…” has prevented you to visit/be with your relatives/friends?	1	2	3	4	5
Preventive Behaviors	13. To what extent the risk of “…” has affected your safety/health behaviors?	1	2	3	4	5
	14. To what extent the risk of “…” has affected your use of sanitizers/detergents?	1	2	3	4	5
	15. To what extent the risk of “…” has affected you to keep sanitizers/detergents available/in your disposal?	1	2	3	4	5
